# Smoking Cessation after Diagnosis of New-Onset Atrial Fibrillation and the Risk of Stroke and Death

**DOI:** 10.3390/jcm10112238

**Published:** 2021-05-21

**Authors:** So-Ryoung Lee, Eue-Keun Choi, Jin-Hyung Jung, Kyung-Do Han, Seil Oh, Gregory Y. H. Lip

**Affiliations:** 1 Department of Internal Medicine, Seoul National University Hospital, Seoul 03080, Korea; minerva1368@gmail.com (S.-R.L.); seil@snu.ac.kr (S.O.); 2 Department of Internal Medicine, Seoul National University College of Medicine, Seoul 03080, Korea; Gregory.Lip@liverpool.ac.uk; 3 Department of Medical Statistics, College of Medicine, Catholic University of Korea, Seoul 06591, Korea; jungjin115@naver.com; 4 Statistics and Actuarial Science, Soongsil University, Seoul 06978, Korea; hkd917@naver.com; 5 Liverpool Centre for Cardiovascular Science, University of Liverpool and Liverpool Chest & Heart Hospital, Liverpool L14 3PE, UK; 6 Department of Clinical Medicine, Aalborg University, 9000 Aalborg, Denmark

**Keywords:** smoking, atrial fibrillation, stroke, death

## Abstract

Limited data are available regarding the impact of smoking cessation after atrial fibrillation (AF) diagnosis on clinical outcomes. Using the Korean National Health Insurance Service database, we included patients newly diagnosed with AF and categorized them into four groups as follows: (i) never smokers, (ii) ex-smokers, (iii) smoking cessation after AF diagnosis (“quitters”), and (iv) current smokers. The primary outcomes were incident ischemic stroke and all-cause death during follow-up. Fatal ischemic stroke and death from cerebrovascular events were evaluated as secondary outcomes. Among 97,637 patients (mean age, 61 years; mean CHA_2_DS_2_-VASc score, 2.3), 6.9% stopped smoking after AF diagnosis. The mean follow-up duration was 3.2 ± 2.0 years. After multivariable adjustment, quitters had lower risks of ischemic stroke (hazard ratio (HR), 0.702; 95% confidence interval (CI), 0.595–0.827) and all-cause death (HR, 0.842; 95% CI, 0.748–0.948) than current smokers. Quitters after AF diagnosis were associated with lower risks of fatal ischemic stroke (HR, 0.454; 95% CI, 0.287–0.718) and death from cerebrovascular events (HR, 0.664; 95% CI, 0.465–0.949) compared with current smokers. Quitting smoking may reduce the risk of ischemic stroke, the severity of ischemic stroke, and the incidence of cerebrovascular events in patients with new-onset AF.

## 1. Introduction

Atrial fibrillation (AF) is the most commonly encountered cardiac arrhythmia in clinical practice, with an increasing prevalence associated with aging of the population [1,2,3]. The risk of AF is related to concomitant cardiovascular diseases and lifestyle, and the identification and management of these risk factors is important for modifying the risk of AF and the burden of AF-related complications.

Smoking is associated with more than a two-fold increased risk of AF, and quitters showed a lower trend of incident AF compared with current smokers [4]. Although the risk of AF increases, smoking also increases the risk of stroke in the general population [5,6]. Previous studies have also shown an association between smoking and an increased risk of stroke and death in patients with AF [7,8].

AF per se increases the risk of stroke by 5-fold and the risk of all-cause death by 1.9-fold in men and by 1.5-fold in women [9,10], consistent with recent studies [3,11,12]. Stroke is the most common cause of death in patients with AF, and AF-related stroke is a considerable healthcare burden [12,13]. Thus, stroke prevention with oral anticoagulation (OAC) therapy is a cornerstone of AF management [14,15,16], and the identification and management of modifiable clinical risk factors is crucial for improving clinical outcomes in patients with incident AF. However, there are limited data on changing lifestyle behaviors, such as smoking cessation after AF diagnosis, on clinical outcomes. Based on the lack of evidence, current guidelines largely underemphasize the importance of smoking cessation in the management of patients with AF [14,15,16]. Therefore, we aimed to evaluate the association between smoking cessation after newly diagnosed AF and the risk of ischemic stroke and all-cause death.

## 2. Materials and Methods

We used the Korean National Health Insurance Service (NHIS) database to acquire the data used in this study [17]. Approximately 50 million people (the entire Korean population) are provided with mandatory health insurance coverage by the government. The Korean NHIS database includes demographic information and information on medical expenses of enrollees, diagnoses, examinations, prescription dispensing records, and procedures for inpatient and outpatient services [17,18]. Diagnoses are coded using the International Classification of Diseases, Tenth Revision, Clinical Modification codes. Furthermore, all enrollees aged ≥40 years are recommended to undergo biannual health screening provided by the Korean National Health Insurance Corporation. The health examination includes anthropometric measurement, blood pressure, laboratory examination, chest X-ray, and a self-reported questionnaire on health behavior, including smoking, alcohol consumption, and physical activity. The participation rate was 75% in 2014 [18]. These data were linked to the Korean NHIS database as a National Health Screening database. All data generated and analyzed during the current study are available from the National Health Insurance Data Sharing Service (accessed at http://nhiss.nhis.or.kr/bd/ab/bada000eng.do (accessed on 1 April 2021)).

This study complied with the principles of the Declaration of Helsinki. This study was exempt from review by the Institutional Review Board of Seoul National University Hospital (E-2003-027-1106).

### 2.1. Study Design and Study Population

**Figure 1:**
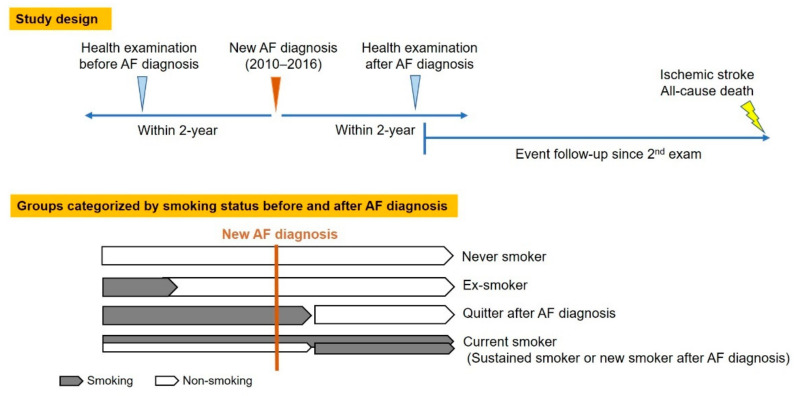
Study design. AF: Atrial fibrillation.

**Figure 2:**
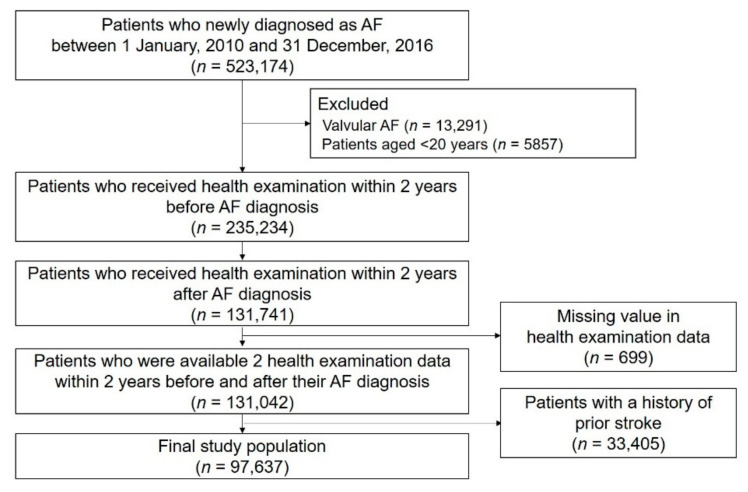
Study enrollment flow. AF: Atrial fibrillation.

The study design and enrollment flow are presented in Figure 1 and Figure 2. We identified 523,174 patients who were newly diagnosed with AF between 1 January 2010 and 31 December 2016. Patients who underwent health examinations both within 2 years prior to AF diagnosis and within 2 years after AF diagnosis were included. Patients diagnosed with valvular AF and those <20 years were excluded. Patients with missing value(s) in health examination data were excluded. To enhance accuracy in the diagnosis of stroke as a clinical outcome, we excluded patients with a history of stroke before the second health examination. Detailed definitions of diagnoses, such as AF, valvular AF, and prior stroke, are shown in Table S1 [2,17].

Study patients were categorized by smoking status and changes before and after AF diagnosis (Figure 1). Smoking status was evaluated using a self-reported questionnaire in the first and second health examinations [19,20,21,22,23,24]. In this self-reported questionnaire, subjects chose one answer among “never smokers,” “ex-smokers”, and “current smokers” and recorded the cumulative amount of their smoking as packs per year. Patients were categorized into four groups based on two sequential questionnaires: never smokers, ex-smokers, quitters after AF diagnosis, and current smokers. Never smokers were defined as patients who had never smoked; ex-smokers were defined as patients who had quit smoking before the first examination and who had sustained their nonsmoking status until the second examination (namely, ex-smokers were those who stopped smoking at any time point before their incident AF); quitters were defined as patients who were current smokers in the first health examination but who quit smoking after AF diagnosis by the time of the second examination; current smokers were defined as patients who were current smokers in the second examination, regardless of smoking status in the first examination.

### 2.2. Covariates

Covariates included patients’ demographic data, comorbidities, medications, and results of health examinations. Patient demographic data, comorbidities, and medications were obtained from the Korean NHIS database. Table S1 summarizes the definitions of the comorbidities [2,17]. Comorbidities included hypertension, diabetes mellitus, dyslipidemia, heart failure, prior myocardial infarction (MI), peripheral artery disease (PAD), chronic kidney disease (CKD), chronic obstructive pulmonary disease (COPD), and cancer [2,25,26,27]. The CHA_2_DS_2_-VASc score for assessing stroke risk was calculated by combining covariate information (Table S1) [28]. Baseline medications, including OACs (i.e., warfarin or direct oral anticoagulants, DOACs), aspirin, P2Y12 inhibitors, and statins, were identified between the date of AF diagnosis and the date of the second health examination. Baseline variables such as body mass index, waist circumference, systolic and diastolic blood pressure, fasting glucose, total cholesterol, low-density lipoprotein cholesterol, high-density lipoprotein cholesterol, and estimated glomerular filtration rate at the second health examination were collected. Alcohol consumption was categorized as nondrinker, mild-to-moderate drinker (>0 g/day to <30 g/day), and heavy drinker (≥30 g/day). Regular exercise was defined as performing >30 min of moderate physical activity ≥5 times a week, or >20 min of strenuous physical activity ≥5 times per week. Low income was defined as patients with an income in the lower 20% of the entire Korean population of subjects supported by the Medical Aid program.

### 2.3. Study Outcomes and Follow-Up

The date of the second health examination was defined as the index date (Figure 1). Incident ischemic stroke and all-cause death were identified as the primary outcome events. The ischemic stroke included all the etiologies of ischemic stroke, including embolic stroke. The secondary outcome events were fatal ischemic stroke and death from cerebrovascular events. The detailed definitions of the study outcomes are presented in Table S1 [17,29]. Patients were followed up from the index date until the occurrence of ischemic stroke, death, or 31 December 2017, whichever came first (Figure 1).

### 2.4. Statistical Analysis

Baseline characteristics are presented as mean ± standard deviation for continuous variables and as numbers (percentages) for categorical variables. To evaluate the significance of the differences among groups categorized by smoking status, one-way analysis of variance and the chi-square test were used. The incidence rates of ischemic stroke and all-cause death were calculated by dividing the number of incident cases by the total follow-up period and presented as per 1000 person-years. Hazard ratios (HRs) and 95% confidence intervals (CIs) for ischemic stroke and all-cause death were analyzed using the Cox proportional hazards model. The HR is an estimate of the ratio of the hazard rate in the comparators versus the reference group [30]. The assumption of proportional hazards regression is that HR is constant over time. Thus, the HR indicates the relative likelihood of the occurrence of the clinical event in comparators versus reference group at any given time point. First, we provided an unadjusted HR. Then, a multivariable-adjusted proportional hazards model was applied; model 1 was adjusted for age and sex and model 2 was adjusted for age, sex, comorbidities including hypertension, diabetes mellitus, dyslipidemia, heart failure, prior MI, PAD, CKD, COPD, and cancer; baseline medications including OACs, aspirin, P2Y12 inhibitor and statin, baseline body mass index, heavy drinker, regular exercise, low income, and CHA_2_DS_2_-VASc score.

Models 1 and 2, which were adjusted sequentially based on a priori considerations for baseline covariates, were also evaluated (Models 3 to 6). Model 3 included age, sex, baseline body mass index, heavy drinking, regular exercise, low income, and CHA_2_DS_2_-VASc score; model 4 included all variables in model 3 except CHA_2_DS_2_-VASc score and added comorbidities including hypertension, diabetes mellitus, dyslipidemia, heart failure, and prior MI and PAD; Model 5 included all variables in Model 4 and added CKD, COPD, and cancer; Model 6 included all variables in model 5 and added CHA_2_DS_2_-VASc score.

Additionally, early rhythm control could affect the incidence of clinical outcomes [31], and we evaluated and further adjusted for the use of antiarrhythmic drugs and those undergoing AF ablation. Obstructive sleep apnea could also affect the risk of ischemic stroke [32]. We evaluated and further adjusted for the prevalence of obstructive sleep apnea in this study.

HRs are presented with never smokers as the reference group (model 2) and also presented with current smokers as the reference group (model 2*) to more intuitively present the relative risk of each group compared with current smokers. To account for the possible influence of the cumulative smoking amount, we stratified each smoking group by smoking amount into 0–10, 10–20, 20–30, and ≥30 pack-years. The potential effect modification by sex, age (<65 years, 65–74 years, and ≥75 years), patients’ underlying stroke risk assessed by CHA_2_DS_2_-VASc score (<3 and ≥3), and OAC treatment status (non-OAC and OAC) were evaluated using subgroup analyses and interaction tests. *p* for interaction <0.1 was considered the presence of significant interaction. Statistical significance was set at *p* < 0.05. For statistical analyses, we used SAS version 9.4 (SAS Institute, Cary, NC, USA).

## 3. Results

We included 97,637 patients in the analysis (mean age, 61.3 ± 12.3 years; men, 62.3%; mean CHA_2_DS_2_-VASc score, 2.3 ± 1.5) (Figure 2). The mean time intervals between the first health exam and AF diagnosis, between AF diagnosis and the second health exam, and between the first and second health exams were 1.1 ± 0.5, 0.9 ± 0.5, and 2.0 ± 0.5 years, respectively. Of the study population, 51.2% were never smokers, 27.3% were ex-smokers, 6.9% were quitters after AF diagnosis, and 14.6% were current smokers. The baseline characteristics of the study population are presented in Table 1. Compared to never smokers, the mean age and CHA_2_DS_2_-VASc score of ex-smokers, quitters after AF diagnosis, and current smokers gradually became younger and lower, respectively. The prevalence of comorbidities, including hypertension, diabetes, heart failure, prior MI, COPD, and cancer, was higher in quitters after AF diagnosis than in the other groups. Moreover, the rate of OAC treatment was slightly higher in quitters after AF diagnosis than in the other groups. Heavy drinkers were more prevalent in current smokers than in the other groups. Among all smokers, the total smoking (packs/year) was highest in quitters after AF diagnosis, followed by current smokers and ex-smokers.

### 3.1. Primary Outcomes

#### 3.1.1. Ischemic Stroke and All-Cause Death Stratified by Smoking Status

**Figure 3:**
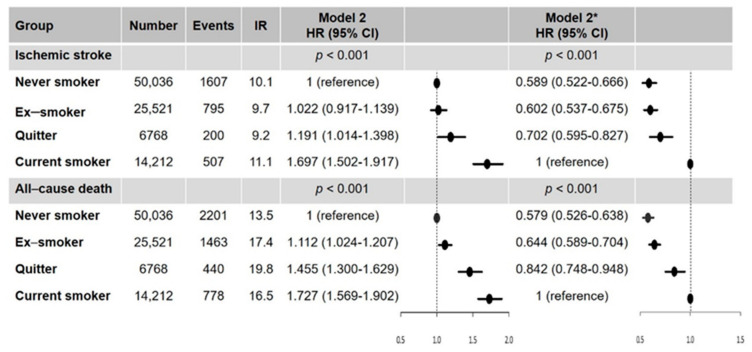
Hazard ratios of smoking status on the risk of ischemic stroke and all-cause death. * With current smokers as the reference group; IR, per 1000 person-years. Abbreviation: CI, confidence interval; HR, hazard ratio; IR, incidence rate.

During a mean follow-up duration of 3.2 ± 2.0 years, a total of 3109 ischemic strokes and 4882 all-cause death events occurred (10.0 per 1000 person-years and 15.4 per 1000 person-years, respectively). The crude event numbers, incidence rates, and HRs for ischemic stroke and death categorized by smoking status are presented in Table S2 and Figure 3.

In multivariable analyses (model 2), ex-smokers were not found to have a higher risk of ischemic stroke compared with never smokers. Smoking cessation after AF diagnosis (quitters after AF diagnosis) and current smokers had a higher risk of ischemic stroke than never smokers (quitters: HR, 1.191; 95% CI, 1.014–1.398; *p* = 0.032; current smokers: HR, 1.697; 95% CI, 1.502–1.917; *p* < 0.001). With current smokers as the reference, quitters after AF diagnosis had a significantly lower risk of ischemic stroke (HR, 0.702; 95% CI, 0.595–0.827; *p* < 0.001). Never smokers and ex-smokers also had lower risks of ischemic stroke than current smokers (never smokers: HR, 0.589; 95% CI, 0.522–0.666; *p* < 0.001; ex-smoker: HR, 0.602; 95% CI, 0.537–0.675; *p* < 0.001) (Figure 3).

For all-cause death, compared to never smokers, smokers were associated with a higher risk of all-cause death with different impacts. The risk of all-cause death was higher in ex-smokers (HR, 1.112; 95% CI, 1.024–1.207; *p* = 0.011), quitters after AF diagnosis (HR, 1.455; 95% CI, 1.300–1.629; *p* < 0.001), and current smokers (HR, 1.727; 95% CI, 1.569–1.902; *p* < 0.001). With current smokers as the reference, quitters after AF diagnosis had a lower risk of all-cause death (HR, 0.842; 95% CI, 0.748–0.948; *p* = 0.005). Never smokers and ex-smokers also had a lower risk of all-cause death than current smokers (never smokers: HR, 0.579; 95% CI, 0.526–0.638; *p* < 0.001; ex-smoker: HR, 0.644; 95% CI, 0.589–0.704; *p* < 0.001) (Figure 3).

Results were consistent in different multivariable adjustment models (Figure S1). When we additionally adjusted for the use of antiarrhythmic drugs and those undergoing AF ablation, the results remained consistent (Tables S3 and S4). After adjusting for the presence of obstructive sleep apnea, the results remained consistent (Tables S5 and S6).

#### 3.1.2. Subgroup Analyses by Sex, Age, and CHA_2_DS_2_-VASc Score

In the subgroup analysis by sex, the results were consistent with the main results in men (Figure S2). With current smokers as the reference, quitters after AF diagnosis had a significantly lower risk of ischemic stroke (HR, 0.677; 95% CI, 0.569–0.806; *p* < 0.001) and all-cause death (HR, 0.869; 95% CI, 0.768–0.082; *p* = 0.023) (Figure S2, panel A). In women, although each smoker group category (except for never smokers) included a relatively small number of patients, quitters after AF diagnosis and ex-smokers showed no significant difference in the risk of ischemic stroke compared to current smokers (Figure S2, A panel). For all-cause death, the results were consistent with the main results (Figure S2, B panel). The *p*-values for the interaction between sex and the impact of smoking status on the risk of ischemic stroke and all-cause death were 0.102 and 0.016, respectively.

In the subgroup analysis by age group (<65 years, 65–74 years, and ≥75 years), although the overall results were consistent with the main results, the *p*-values for interaction between the age subgroups and the impact of smoking status on the risk of ischemic stroke and all-cause death were both <0.001 (Figure S3, A panel). In patients aged 65–74 years, quitters after AF diagnosis had a significantly lower risk of ischemic stroke than current smokers (HR, 0.546; 95% CI, 0.411–0.725; *p* < 0.001), whereas the other age subgroups did not show a significantly lower risk in the quitter after AF diagnosis group.

The impact of quitting smoking compared with current smoking was slightly more attenuated in patients with a CHA_2_DS_2_-VASc score ≥3 (HR, 0.769; 95% CI, 0.605–0.979) than in those with a CHA_2_DS_2_-VASc score <3 (HR, 0.649; 95% CI, 0.515–0.816) (Figure S3, B panel). The trends of the impact of smoking status on the risk of ischemic stroke and all-cause death were consistent in both the CHA_2_DS_2_-VASc subgroups and the main results. A significant interaction was observed in both ischemic stroke (*p* = 0.027) and all-cause death (*p* < 0.001) because of the difference in impact size.

#### 3.1.3. Impact of Smoking Amount on the Risk of Ischemic Stroke and All-Cause Death

When stratifying each smoker group by smoking amount (0–10, 10–20, 20–30, and ≥30 pack-years), ex-smokers were not found to have a higher risk of ischemic stroke compared with never smokers. In contrast, current smokers were consistently associated with a higher risk of ischemic stroke compared with never smokers (Table 2). Among quitters after AF diagnosis, patients with <30 packs per year were not associated with a higher risk of ischemic stroke compared with never smokers. Quitters after AF diagnosis with ≥30 packs per year had a higher risk of ischemic stroke compared with never smokers; however, the HR was lower than that of current smokers, regardless of the smoking amount. For all-cause death, ex-smokers with <30 packs per year were not associated with an increased risk of all-cause death compared with never smokers. Quitters after AF diagnosis and current smokers had a higher risk of all-cause death than never smokers; generally, the HRs of current smokers were higher than those of quitters after AF diagnosis, regardless of smoking amount.

### 3.2. Secondary Outcomes: Fatal Ischemic Stroke and Death from Cerebrovascular Events Stratified by Smoking Status

**Figure 4:**
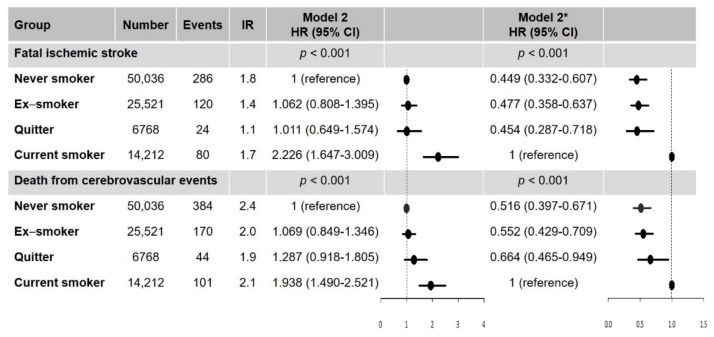
Hazard ratios of smoking status on the risk of fatal ischemic stroke and fatal cerebrovascular event. * With current smokers as the reference group. IR, per 1000 person-years. Abbreviation: CI, confidence interval; HR, hazard ratio; IR, incidence rate.

Among the four groups, current smokers had a higher risk of fatal ischemic stroke and death from cerebrovascular events than the other groups (Figure 4). Quitters after AF diagnosis also had a significantly lower risk of fatal ischemic stroke (HR, 0.454; 95% CI, 0.287–0.718) and death from cerebrovascular events (HR, 0.664; 95% CI, 0.465–0.949) compared with current smokers (Figure 4).

### 3.3. Clinical Outcomes in Different Smoking Status According to the OAC Treatment

**Figure 5:**
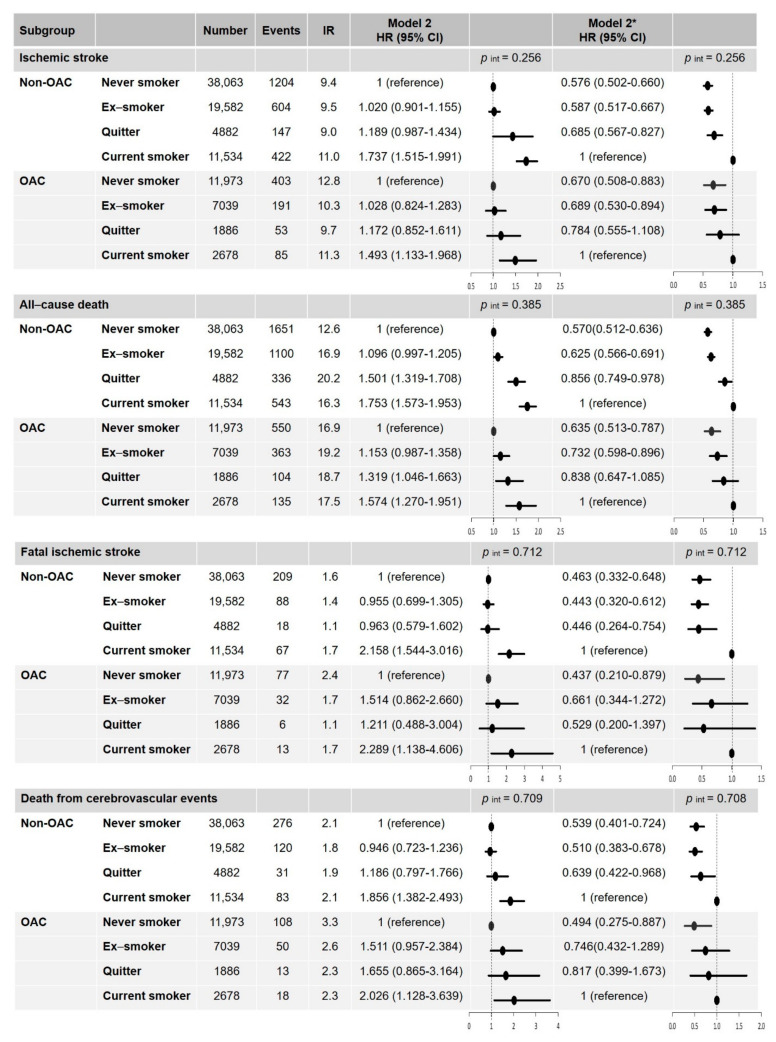
Hazard ratios of smoking status on the risk of ischemic stroke, fatal ischemic stroke, death from cerebrovascular events, and all-cause death according to their OAC treatment status. * With current smokers as the reference group. IR, per 1000 person-years. Abbreviation: CI, confidence interval; HR, hazard ratio; IR, incidence rate; OAC, oral anticoagulation; *p* int, *p* for interaction.

In the subgroup without OAC treatment, the results were consistent with the main results (Figure 5). Although the risk reduction of each clinical outcome was slightly attenuated in the subgroup with OAC treatment, there were no significant interactions between the impact of smoking status on the clinical outcomes and the subgroups when stratified by OAC treatment (Figure 5).

## 4. Discussion

To the best of our knowledge, this is the first and largest study to report the clinical impact of smoking status (and the change in the latter following AF diagnosis) on ischemic stroke and death in patients with newly diagnosed AF. The main findings were as follows: (i) a substantial proportion of patients who were newly diagnosed with AF remained as current smokers even after their AF diagnosis (14.6%), whereas 6.9% of patients stopped smoking after their AF diagnosis; (ii) current smoking was associated with the highest risk of ischemic stroke and all-cause death; (iii) compared with current smokers, quitters after AF diagnosis had a lower risk of ischemic stroke by 30% and all-cause death by 16%; (iv) compared with current smokers, quitters had a significantly lower risk of fatal ischemic stroke by 55% and death from cerebrovascular events by 34%. This suggests that the benefit of risk reduction of all-cause death in quitters is mainly caused by the reduction in fatal neurologic adverse events; (v) the benefit of smoking cessation after AF diagnoses on the risk reduction of primary and secondary clinical outcomes was significant, irrespective of the OAC treatment status.

Current guidelines have emphasized the importance of appropriate OAC treatment according to patients’ risk profiles to reduce the risk of stroke and improve overall outcomes in patients with AF [14,15,16]. The initial period after AF diagnosis is critical for three reasons. First, the risk of clinical events in patients with AF appears to be especially higher than that in patients without AF within the initial period after diagnosis [3]. In large observational studies, the HR for ischemic stroke was 13.28 (95% CI, 10.89–16.20) within 6 months after AF diagnosis and decreased after 6 months (HR, 3.31; 95% CI, 3.23–3.39) when compared to non-AF patients in each period [3]. Second, risk assessment for stroke should be completed to determine optimal OAC treatment [14,15,16]. Third, in the initial period of AF diagnosis, motivating patients to adopt a healthy lifestyle can be more impactful and implementable to patients. Although suggesting a healthy lifestyle to achieve better clinical outcomes is an important part of primary prevention for patients with AF, current AF guidelines have not emphasized this topic due to lack of evidence.

The implementation of adequate OAC therapy for stroke prevention, better control of AF symptoms, and management of concomitant comorbidities and cardiovascular risk factors are crucial for the optimal care of patients with AF. Among these, lifestyle modification is part of the management of concomitant cardiovascular risk [33]. Although there is some evidence of the clinical impact of lifestyle modifications in patients with AF, modifying behavior is still underemphasized in recent guidelines. For example, in the 2016 European Society of Cardiology Guidelines for AF management [34], there was just one recommendation, as follows: “patient involvement in the care process should be considered to encourage self-management and responsibility for lifestyle changes” (Class IIA, Level of Evidence C). Although smoking is mentioned as a risk factor for stroke in patients with AF in more recently updated guidelines, there have been no specific recommendations for smoking. In contrast, other lifestyle modifications, such as weight loss, blood pressure control, and alcohol reduction, are generally emphasized more in the currently updated guidelines [15]. To educate and encourage smoking cessation in patients who are newly diagnosed with AF, more data are needed to inform evidence-based guideline recommendations. Based on the results of the present study, smoking cessation should be added to the “to-do list” after AF diagnosis.

Previous studies have reported the impact of smoking status on cardiovascular outcomes in patients with AF [7,8,35]. One clinical cohort trial suggested that smoking may increase the risk of thromboembolic events in patients with anticoagulated AF by 2-fold [7]. Of note, smoking has not been included in stroke risk stratification schemes [14,15,16], and one previous study failed to demonstrate that smoking was a significant and independent risk factor for stroke in patients with AF [36]. In the Danish Diet, Cancer and Health cohort study, which included 3161 patients with incident AF, smoking did not show a significant increase in the risk of stroke in both men and women; however, this study did not include an analysis of the impact of quitting smoking after AF diagnosis [8]. Another recent study evaluated the association between smoking habits and the incidence of cardiovascular outcomes in patients with AF [35]. Although this study showed similar results to those of the current study, our study has several additional strengths. First, we included a substantially larger population cohort (97,637 vs. 2372), including women; thus, the conclusion can be interpreted as being more generalizable. In addition, based on a much larger scale study population, we were able to analyze the consistency of the main results in various subgroups and to evaluate the dose–response relationship between smoking amount and clinical outcomes. Second, the previous report evaluated various clinical outcomes, including composite cardiovascular disease, stroke, coronary heart disease, and acute MI, and the results showed significant reduction in the risk of composite cardiovascular disease, total stroke, and ischemic stroke. In the present analysis, we focused more on ischemic stroke and added all-cause death as a relevant hard endpoint (to access net clinical benefit). To address the risk reduction of all-cause death in “quitters after AF diagnosis” and the impact of quitting smoking on the severity of ischemic stroke, we added data on fatal stroke events and deaths from cerebrovascular disease as secondary outcomes. Based on previous studies, the clinical impact of smoking on the severity of stroke remains controversial and has been reported to vary in different etiologies of stroke [37,38,39]. In the present analysis, we demonstrate that not only was the risk of ischemic stroke lower, but the risks of fatal ischemic stroke and death from cerebrovascular events were also significantly lower in quitters after AF diagnosis than in current smokers. Third, the study period differed between the two studies. The previous report by Choi et al. [35] analyzed data from 2003 to 2012, when warfarin was the only option for OAC therapy and OAC prescription was low (approximately 15% of the total population). In the present study, we included patients from 2010 to 2016 with a much higher OAC treatment rate (approximately 25% of the total population), including 7905 patients treated with DOACs. The present study therefore reflects a much more contemporary treatment pattern of OAC, which is an important confounder when considering ischemic stroke. In the subgroup analysis by OAC treatment status, the main results showing that quitters after AF diagnosis had lower risks of clinical outcomes compared with current smokers were consistently observed in both the non-OAC and OAC groups. Although the beneficial effect of quitting smoking was slightly attenuated in the OAC groups, the importance of quitting smoking should still be emphasized to all patients with AF, regardless of OAC treatment. Even though we included a larger number of patients treated with DOAC than in previous studies [8,35], warfarin was still mainly used in this study population. The proportion of DOAC use was limited. Therefore, the impact of wider use of DOAC, as currently observed in many countries, is unknown.

In the general population, smoking is a well-established risk factor associated with an increased risk of stroke [6,40]. In addition, the Framingham study demonstrated that quitting cigarette smoking led to a lower risk of stroke compared with continued smoking, but there was no significant difference between quitters and nonsmokers with regard to stroke risk [6]. The quitting effect occurred quite soon after smoking cessation, and the risk of stroke had decreased significantly 2 years after quitting and became similar to that of nonsmokers within 5 years of quitting [6].

Our study showed results consistent with those of a previous population-based study among patients with AF. Quitters after AF diagnosis showed a higher prevalence of comorbidities than that of other groups at the baseline. There is the possibility that more proactive lifestyle modification might be implemented in so-called “sicker patients” at baseline. After adequately adjusting for differences in the baseline characteristics, quitters after AF diagnosis were significantly associated with lower risks of ischemic stroke, all-cause death, fatal ischemic stroke, and death from cerebrovascular disease. The rapid risk reduction after quitting smoking can be explained by the possible mechanisms by which smoking precipitates thromboembolic events by increasing serum fibrinogen levels, promoting a hypercoagulable state, and causing platelet dysfunction [41,42,43,44,45]. Although increased thrombogenicity is associated with an increased risk of stroke in current smokers, as in the general population, there is limited evidence of the role of AF in the risk reduction of ischemic stroke after smoking cessation. The effect of aggressive risk factor reduction, including smoking cessation after AF ablation, has been reported [46]. In this previous observational study, aggressive risk factor management reduced AF recurrence after AF ablation. Lifestyle factors may be the primary promoters of AF substrates to facilitate rhythm control strategies; however, this previous study included a relatively small number of patients and elucidated the effect of the “cluster” of risk factor management rather than each individual risk factor. Further investigation is therefore needed to explain the mechanism of stroke risk reduction after smoking cessation, especially the role of the AF burden.

Unfortunately, this study did not include a detailed etiology of ischemic stroke given the inherent limitations of the database, which lacks detailed results of brain imaging studies. Although it is well known that cardiogenic embolism is a common cause of ischemic stroke in patients with AF, other subtypes of ischemic stroke are also responsible for a substantial proportion of strokes in patients with AF [47,48,49]. In addition, patients with higher CHA_2_DS_2_-VASc scores have a higher chance of having more risk factors for both cardioembolic and non-cardioembolic ischemic stroke [50]. Hence, to reduce the overall ischemic stroke risk burden in patients with AF, a general reduction in overall cardiovascular risk is also necessary. The remaining risk after smoking cessation might be mediated by atherosclerosis, which will impact long-term outcomes, particularly the risk of all-cause mortality [51,52]. Of note, smoking cessation was favorable for the ischemic stroke outcome in patients with a substantial cumulative smoking amount, but the benefit was attenuated in prior heavy smokers (≥30 pack-years) among quitters after AF diagnosis (Table 2). The attenuation of benefit in prior heavy smokers could be related to long-term vascular and endothelial damage associated with atherothrombosis.

We observed several interactions between different subgroups and the impact of smoking status on the risk of ischemic stroke and all-cause death. However, the statistical power was affected by the total number of patients and the incidence rate of clinical outcomes in each subgroup. Although the benefit of quitters after AF diagnosis was more accentuated in a certain subgroup, such as men, patients aged 65–74 years, and those with lower CHA_2_DS_2_-VASc scores, it is important to consider that the crude incidence rates of ischemic stroke and all-cause death varied widely among the different subgroups. In addition, the total number of certain subgroups was relatively smaller than in others (e.g., women and patients aged ≥ 75 years). These factors should be considered when interpreting the results of subgroup analyses. Despite the fact that the HRs were slightly attenuated and the CI of quitters after AF diagnosis became wider in a certain subgroup, the directionality of the HRs of quitters after AF diagnosis was consistent with the main results. However, further studies on the impact of quitting smoking in the women subgroup or patients at a lower risk of stroke are needed in the future.

### Limitations

Our study has several limitations. First, this was an observational, retrospective cohort study, and therefore, the relationship between smoking cessation and lower risks of ischemic stroke and death is limited to association rather than causality. Considering the ethical issues of the lifestyle modification study (e.g., randomizing cardiac patients to keep smoking or stopping smoking), we assumed that randomized clinical trials could not be performed to support this hypothesis. Second, although we considered and carefully adjusted covariates that could affect the clinical outcomes, there is a possibility that confounding factors have remained. For the given variables, we applied a stepwise approach to gradually adding clinical variables. The main results were consistently observed in various multivariable adjustment models (Figure S1). Third, although this study included a large population, only Asians from a single nationality were included, which should be considered when generalizing the findings. Fourth, we excluded patients with a history of stroke. Patients who experienced a stroke before AF diagnosis might have a greater burden of atherosclerotic disease than those who did not [53], and the self-motivation or reinforcement for lifestyle modification (i.e., quitting smoking) by physicians might be different. Fifth, the prescription of OACs was relatively low, but this has been consistently observed in previous reports from Asian real-world practice [54,55]. More optimal OAC therapy after a diagnosis of AF is necessary to reduce the risk of stroke. Despite these limitations, we have analyzed the largest number of study populations to generate the persuasive main recommendation that smoking status should become an important lifestyle factor to be routinely considered and addressed in the management of patients presenting with the first episode of AF. Sixth, since the estimation of smoking habits was based on the self-reported questionnaires, there might be a possibility of underestimating the true prevalence of smoking. Although this study did not include the measurement of exhaled carbon monoxide levels, it could be useful to evaluate actual smoking status [56]. Seventh, there is a possibility that the dynamic changes of other lifestyle factors could affect the risk of clinical outcomes. We adjusted for comorbidities, physical activity, body mass index, and alcohol intake assessed at the second health examination (the latest status, namely, at the starting point of clinical events follow-up) in the multivariable adjustment model. Since the aim of the study was to evaluate the impact of smoking status changes on the risk of ischemic stroke and all-cause death in patients with incident AF, we did not analyze the impact of changes in several other lifestyle factors on the clinical outcomes. Eighth, although the type, the burden, or the recurrence of AF could affect clinical outcomes, we did not have detailed information on these factors given the inherent limitations of the database. Further studies are needed on the type or burden of AF, which would be a possible mediator for the increased risk of ischemic stroke and all-cause death in current smokers. Lastly, we only focused on patients with AF in this study, because these patients have a higher risk of stroke. In patients with AF, the etiology is more likely to be cardioembolic stroke, and the risk of cardiovascular and all-cause death is also much higher [12]. In a recent report from our group, even among AF patients at low risk of stroke (CHA_2_DS_2_-VASc score 0 in men or 1 in women), current smoking was the only independent and significant factor for predicting ischemic stroke due to cardioembolic mechanisms [57]. Therefore, we believe that it is worthwhile and necessary to evaluate the risk of smoking on stroke and the beneficial effects of quitting smoking on reducing stroke risk in special populations such as patients with AF. The results of this study could provide evidence for holistic management, including lifestyle modification beyond medical therapy alone for patients with AF.

## 5. Conclusions

Smoking cessation after incident AF was associated with a lower risk of ischemic stroke and all-cause death compared with current smoking. Lifestyle changes, such as quitting smoking after a diagnosis of AF, may reduce the risk of ischemic stroke and reduce the severity of ischemic stroke and cerebrovascular events in patients with new-onset AF.

## Figures and Tables

**Table 1:** Baseline characteristics of the total study population by smoking status.

	Total (*n* = 97,637)	Never Smoker (*n* = 50,036)	Ex-Smoker (*n* = 26,621)	Quitter (*n* = 6768)	Current Smoker (*n* = 14,212)	*p*-Value
**Age (years)**	61.3 ± 12.3	63.1 ± 12.0	62.2 ± 11.2	57.8 ± 12.2	54.8 ± 12.4	<0.001
**<65 years**	54,735 (56.1)	24,667 (49.3)	14,538 (54.6)	4622 (68.3)	10,908 (76.8)	<0.001
**65 to <75 years**	29,458 (30.2)	16,792 (33.6)	8472 (31.8)	1627 (24.0)	2567 (18.1)	
**≥75 years**	13,444 (13.8)	8577 (17.1)	3611 (13.6)	519 (7.7)	737 (5.2)	
**Sex**						
**Male**	60,839 (62.3)	15,109 (30.2)	25,941 (97.5)	6337 (93.6)	13,452 (94.7)	<0.001
**CHA_2_DS_2_-VASc**	2.33 ± 1.47	2.74 ± 1.5	2.02 ± 1.33	1.97 ± 1.34	1.65 ± 1.24	<0.001
**0**	7908 (8.1)	1809 (3.6)	2949 (11.1)	813 (12.0)	2337 (16.4)	<0.001
**1**	24,061 (24.6)	9523 (19.0)	7466 (28.1)	1965 (29.0)	5107 (35.9)	
**≥2**	65,668 (67.3)	38,704 (77.4)	16,206 (60.9)	3990 (59.0)	6768 (47.6)	
**Comorbidities**						
**Hypertension**	63,297 (64.8)	31,985 (63.9)	17,720 (66.6)	4611 (68.1)	8981 (63.2)	<0.001
**Diabetes mellitus**	20,529 (21.0)	9785 (19.6)	5994 (22.5)	1617 (23.9)	3133 (22.0)	<0.001
**Dyslipidemia**	10,512 (10.8)	5455 (10.9)	2514 (9.4)	783 (11.6)	1760 (12.4)	<0.001
**Heart failure**	23,622 (24.2)	12,279 (24.5)	6433 (24.2)	1924 (28.4)	2986 (21.0)	<0.001
**Prior MI**	4519 (4.6)	1992 (4.0)	1303 (4.9)	587 (8.7)	637 (4.5)	<0.001
**PAD**	19,150 (19.6)	10,511 (21.0)	5035 (18.9)	1224 (18.1)	2380 (16.8)	<0.001
**CKD**	12,779 (13.1)	7632 (15.3)	3261 (12.3)	797 (11.8)	1089 (7.7)	<0.001
**COPD**	18,082 (18.5)	9225 (18.4)	4998 (18.8)	1564 (23.1)	2295 (16.2)	<0.001
**Cancer**	5679 (5.8)	2900 (5.8)	1733 (6.5)	641 (9.5)	405 (2.9)	<0.001
**Medication**						
**OAC**	23,576 (24.2)	11,973 (23.9)	7039 (26.4)	1886 (27.9)	2678 (18.8)	<0.001
**Warfarin**	17,341 (16.1)	7549 (15.1)	4694 (17.6)	1426 (21.1)	2002 (14.1)	<0.001
**DOAC**	7905 (8.1)	4424 (8.8)	2345 (8.8)	460 (6.8)	676 (4.8)	<0.001
**Aspirin**	20,482 (21.0)	10,091 (20.2)	6107 (22.9)	1409 (20.8)	2875 (20.2)	<0.001
**P2Y_12_ inhibitor**	4965 (5.1)	2334 (4.7)	1480 (5.6)	501 (7.4)	650 (4.6)	<0.001
**Statin**	15,664 (16.0)	8494 (17.0)	4151 (15.6)	1105 (16.3)	1914 (13.5)	<0.001
**Health exam parameter**						
**BMI (kg/m^2^)**	24.5 ± 3.3	24.5 ± 3.4	24.7 ± 3.1	24.7 ± 3.4	24.5 ± 3.4	<0.001
**Waist circumference (cm)**	84.4 ± 9.5	82.5 ± 10.1	86.6 ± 8.2	86.4 ± 8.8	85.7 ± 8.7	<0.001
**Systolic BP (mmHg)**	125.2 ± 15.3	125.3 ± 15.8	125.7 ± 14.6	124.7 ± 15.1	123.9 ± 14.6	<0.001
**Diastolic BP (mmHg)**	77.0 ± 10.2	76.6 ± 10.3	77.5 ± 10.1	77.3 ± 10.2	77.5 ± 10.2	<0.001
**Fasting glucose (mg/dL)**	104.4 ± 26.7	102.9 ± 25.5	105.5 ± 25.3	106.5 ± 29.4	106.4 ± 31.2	<0.001
**Total cholesterol (mg/dL)**	183.8 ± 41.0	186.1 ± 41.3	179.5 ± 40.0	182.3 ± 41.5	184.7 ± 41.1	<0.001
**LDL-C (mg/dL)**	105.5 ± 41.7	107.8 ± 41.7	102.7 ± 40.7	103.0 ± 38.5	103.7 ± 44.3	<0.001
**HDL-C (mg/dL)**	52.5 ± 15.2	54.0 ± 15.3	51.2 ± 15.8	50.5 ± 13.2	50.5 ± 14.4	<0.001
**eGFR (mL/min/1.73 m^2^)**	81.6 ± 28.0	80.6 ± 27.6	80.5 ± 27.5	82.7 ± 32.1	86.3 ± 27.9	<0.001
**Smoking amount** **(pack-years)**	10.0 ± 15.8	0	19.1 ± 17.4	24.0 ± 19.1	21.4 ± 15.4	<0.001
**0 (never smoker)**	50,036 (51.3)	50,036 (100)	0 (0)	0 (0)	0 (0)	<0.001
**>0 to <10 pack-years**	12,648 (13.0)	0 (0)	8144 (30.6)	1443 (21.3)	3061 (21.5)	
**10 to <20 pack-years**	12,510 (12.8)	0 (0)	6990 (26.3)	1583 (23.4)	3937 (27.7)	
**20 to <30 pack-years**	9181 (9.4)	0 (0)	4863 (18.3)	1301 (19.2)	3017 (21.2)	
**≥30 pack-years**	13,262 (13.6)	0 (0)	6624 (24.9)	2441 (36.1)	4197 (29.5)	
**Alcohol consumption**						
**Nondrinker**	62,450 (64.0)	40,945 (81.8)	13,194 (49.6)	3592 (53.1)	4719 (33.2)	<0.001
**Mild-to-moderate drinker**	28,931 (29.6)	8223 (16.4)	11,005 (41.3)	2562 (37.9)	7141 (50.3)	
**Heavy drinker**	6256 (6.4)	868 (1.7)	2422 (9.1)	614 (9.1)	2352 (16.6)	
**Regular exercise**	21,903 (22.4)	9893 (19.8)	7667 (28.8)	1542 (22.8)	2801 (19.7)	<0.001
**Low income**	16,279 (16.7)	8744 (17.5)	4000 (15.0)	1114 (16.5)	2421 (17.0)	<0.001

Abbreviations: BMI, body mass index; BP, blood pressure; CKD, chronic kidney disease; COPD, chronic obstructive pulmonary disease; DOAC, direct oral anticoagulant; eGFR, estimated glomerular filtration rate; HDL-C, high-density lipoprotein cholesterol; LDL-C, low-density lipoprotein cholesterol; MI, myocardial infarction; OAC, oral anticoagulant; PAD, peripheral artery disease.

**Table 2:** Hazard ratios of ischemic stroke and all-cause death in each group stratified by the smoking amount.

Smoking Status	Smoking Amount (Pack-Years)	Number	Events	IR	Unadjusted HR (95% CI)	Model 1 HR (95% CI)	Model 2 HR (95% CI)
**Ischemic stroke**					<0.001	<0.001	<0.001
**Never smoker**	**0**	50,036	1607	10.0	1 (reference)	1 (reference)	1 (reference)
**Ex-smoker**	**>0 to <10**	8144	198	7.8	0.775 (0.668–0.898)	0.946 (0.805–1.111)	0.957(0.815–1.125)
	**≥10 to <20**	6990	177	8.2	0.812 (0.695–0.948)	0.947 (0.799–1.122)	0.943 (0.795–1.117)
	**≥20 to <30**	4863	149	9.9	0.979 (0.828–1.158)	1.032 (0.861–1.237)	1.038 (0.865–1.244)
	**≥30**	6624	271	13.5	1.335 (1.174–1.519)	1.152 (0.995–1.333)	1.138 (0.982–1.318)
**Quitter**	**>0 to <10**	1443	32	6.8	0.679 (0.479–0.963)	1.164 (0.817–1.657)	1.133 (0.795–1.613)
	**≥10 to <20**	1583	36	7.1	0.703 (0.505–0.977)	1.107 (0.791–1.550)	1.069 (0.764–1.497)
	**≥20 to <30**	1301	34	8.2	0.817 (0.582–1.147)	1.135 (0.803–1.604)	1.087 (0.768–1.537)
	**≥30**	2441	98	12.5	1.247 (1.017–1.529)	1.346 (1.085–1.669)	1.315 (1.059–1.633)
**Current smoker**	**>0 to <10**	3061	90	9.0	0.895 (0.724–1.107)	1.782 (1.429–2.221)	1.745 (1.399–2.177)
	**≥10 to <20**	3937	125	10.0	0.994 (0.829–1.193)	1.802 (1.484–2.190)	1.763 (1.451–2.144)
	**≥20 to <30**	3017	114	11.8	1.173 (0.970–1.418)	1.806 (1.475–2.211)	1.740 (1.419–2.133)
	**≥30**	4197	178	13.1	1.302 (1.116–1.520)	1.667 (1.407–1.976)	1.609 (1.354–1.912)
**All-cause death**					<0.001	<0.001	<0.001
**Never smoker**	**0**	50,036	2201	13.5	1 (reference)	1 (reference)	1 (reference)
**Ex-smoker**	**>0 to <10**	8144	314	12.2	0.906 (0.805–1.020)	0.954 (0.840–1.084)	0.946 (0.833–1.074)
	**≥10 to <20**	6990	312	14.2	1.055 (0.937–1.188)	1.054 (0.927–1.199)	1.050 (0.923–1.193)
	**≥20 to <30**	4863	262	17.0	1.263 (1.111–1.435)	1.099 (0.958–1.261)	1.098 (0.957–1.260)
	**≥30**	6624	575	27.8	2.074 (1.892–2.273)	1.378 (1.242–1.530)	1.293 (1.164–1.436)
**Quitter**	**>0 to <10**	1443	58	12.1	0.901 (0.695–1.170)	1.602 (1.232–2.084)	1.417 (1.089–1.842)
	**≥10 to <20**	1583	85	16.4	1.221 (0.983–1.517)	1.943 (1.557–2.426)	1.592 (1.275–1.987)
	**≥20 to <30**	1301	87	20.6	1.529 (1.234–1.895)	1.971 (1.583–2.455)	1.591 (1.277–1.983)
	**≥30**	2441	210	26.2	1.946 (1.689–2.242)	1.814 (1.561–2.108)	1.377 (1.183–1.601)
**Current smoker**	**>0 to <10**	3061	133	13.0	0.962 (0.807–1.146)	1.938 (1.619–2.320)	1.769 (1.477–2.118)
	**≥10 to <20**	3937	171	13.4	0.992 (0.849–1.159)	1.828 (1.553–2.152)	1.640 (1.392–1.932)
	**≥20 to <30**	3017	169	17.0	1.261 (1.078–1.474)	1.866 (1.583–2.199)	1.713 (1.453–2.021)
	**≥30**	4197	305	21.7	1.607 (1.426–1.812)	1.942 (1.705–2.212)	1.782 (1.562–2.033)

IR, per 1000 person-years. Model 1: age- and sex-adjusted Model 2: age, sex, hypertension, diabetes mellitus, dyslipidemia, heart failure, prior MI, PAD, CKD, COPD, cancer, BMI, heavy drinking, regular exercise, low income, use of OAC, use of antiplatelet agent, use of statin, and CHA_2_DS_2_-VASc score. Abbreviations: BMI, body mass index; CI, confidence interval; CKD, chronic kidney disease; COPD, chronic obstructive pulmonary disease; HR, hazard ratio; IR, incidence rate; MI, myocardial infarction; OAC, oral anticoagulant; PAD, peripheral artery disease.

## Data Availability

All data generated and analyzed during the current study are available at the National Health Insurance Data Sharing Service (accessed at http://nhiss.nhis.or.kr/bd/ab/bada000eng.do (accessed on 1 April 2021).

## Supplementary Materials

The following are available online at https://www.mdpi.com/article/10.3390/jcm10112238/s1, Table S1: Definitions of covariates, Table S2: Hazard ratios of ischemic stroke and all-cause death: unadjusted and model 1, Table S3: The proportion of patients treated with antiarrhythmic drugs and undergoing AF ablation, Table S4: Hazard ratios of primary and secondary outcomes after additionally adjusting whether performing early rhythm control by antiarrhythmic drugs or AF catheter ablation, Table S5: The proportion of patients with obstructive sleep apnea, Table S6: Hazard ratios of primary and secondary outcomes after additionally adjusting for obstructive sleep apnea, Figure S1: Hazard ratios for the risk of ischemic stroke and all-cause death in sequentially adjusted multivariable models; Figure S2: Hazard ratios of smoking status on the risk of ischemic stroke and all-cause death in men and women; Figure S3: Subgroup analyses by age (<65 years, 65–74 years, and ≥75 years) and CHA_2_DS_2_-VASc score (<3 and ≥3).
