# Supplementary material for: Smoking Cessation after Diagnosis of New-Onset Atrial Fibrillation and the Risk of Stroke and Death
Source: J Clin Med. 2021 May 21;10(11):2238. doi: 10.3390/jcm10112238 (PMC8196704; doi:10.3390/jcm10112238)
Supplement: Supplementary file 1 [file jcm-10-02238-s001.zip › jcm-1208735-supplementary.pdf]

Supplementary materials

Online Tables

Online Table S1. Definitions of covariates

Online Table SS2. Hazard ratios of ischemic stroke and all-cause death: unadjusted and model 1

Online Table S3. The proportion of patients treated with antiarrhythmic drugs and undergoing AF ablation

Online Table S4. Hazard ratios of primary and secondary outcomes after additionally adjusting whether performing early rhythm control by antiarrhythmic drugs or AF catheter ablation

Online Table S5. The proportion of patients with obstructive sleep apnea

Online Table S6. Hazard ratios of primary and secondary outcomes after additionally adjusting obstructive sleep apnea

Online Figures

Online Figure S1. Hazard ratios for the risk of ischemic stroke and all-cause death in sequentially adjusted multivariable models

Online Figure S2. Hazard ratios of smoking status on the risk of ischemic stroke and all-cause death in men and women

Online Figure S3. Subgroup analyses by age (<65 years, 65 to 74 years, and ≥75 years) and CHA_2DS_2-VASc score (<3 and ≥3)

Online Tables

Online Table S1. Definitions of covariates

Diagnosis	ICD-10-CM code and definition	Diagnostic definition
**Inclusion/exclusion criteria**		
**Atrial fibrillation**	I48.0-48.4, I48.9	Admission≥1 or outpatient department≥2
**Valvular atrial fibrillation**	I05.0, I05.2, I05.9, Z95.2-Z95.4	Admission or outpatient department≥1
**Ischemic stroke**	I63, I64	Admission or outpatient department≥1
**Comorbidities**		
**Hypertension**	I10-I13, I15; and minimum 1 prescription of anti-hypertensive drug (thiazide, loop diuretics, aldosterone antagonist, alpha-/beta-blocker, calcium-channel blocker, angiotensin-converting enzyme inhibitor, angiotensin II receptor blocker).	Admission≥1 or outpatient department≥2
	or systolic/diastolic blood pressure ≥ 140/90 mmHg	Based on the results of 2nd health exam
**Diabetes mellitus**	E11-E14; and minimum 1 prescription of anti-diabetic drugs (sulfonylureas, metformin, meglitinides, thiazolidinediones, dipeptidyl peptidase-4 inhibitors, α-glucosidase inhibitors, and insulin).	Admission≥1 or outpatient department≥2
	or fasting glucose level ≥ 126 mg/dL	Based on the results of 2nd health exam
**Dyslipidemia**	E78	Admission or outpatient department≥1
	or Total cholesterol ≥ 240 mg/dL	Based on the results of 2nd health exam
**Heart failure**	I50	Admission or outpatient department≥1
**Vascular disease**		
**Prior MI**	I21, I22	Admission or outpatient department≥1
**PAD**	I70, I73	Admission or outpatient department≥2
**CKD**	eGFR<60ml/min/1.73m^2	Based on the results of 2nd health exam
**COPD**	J41-44	Admission≥1
**Cancer**	C00-97 and RID code (V193)	Admission or outpatient department≥1
**Health exam questionnaire**		
**Alcohol consumption**	Mild to moderate drinker: alcohol consumption >0g to <30g per day<br>Heavy drinker: alcohol consumption ≥30g per day	Based on the results of 2nd health exam
**Regular exercise**	Performing a moderate physical activity more than 30 minutes at least 5 times per week or strenuous	Based on the results of 2nd health exam

	physical activity more than 20 minutes at least 3 times per week.	
**Low income**	Income lowest 20% and medical aid	
**Scores**		
**CHA_2DS_2-VASc score**	Heart failure (1 point), hypertension (1 point), age ≥75 years (2 points), diabetes (1 point), previous stroke/systemic embolism/transient ischemic attack (2 points), vascular disease (prior MI or PAD, 1 point) and female sex (1 point)	
**Clinical outcome**		
**Ischemic stroke**	I63, I64	Admission≥1 and brain imaging (CT or MRI) ≥1
**Fatal ischemic stroke**	I63, I64	Ischemic stroke event that led to death within 30-day of its occurrence
**Death from cerebrovascular events**	I60-69	Any cerebrovascular events that led to death within 30-day of its occurrence

Abbreviation: CKD, chronic kidney disease; COPD, chronic obstructive pulmonary disease; CT, computed tomography; eGFR, estimated glomerular filtration rate; MI, myocardial infarction; MRI, magnetic resonance image; PAD, peripheral artery disease.

Online Table S2. Hazard ratios of ischemic stroke and all-cause death: unadjusted and model 1

Smoking status	Unadjusted HR (95% CI)	Model 1 HR (95% CI)
**Ischemic stroke**	P=0.051	P=<0.001
**Never smoker**	1 (reference)	1 (reference)
**Ex-smoker**	0.959 (0.881-1.044)	1.022 (0.917-1.139)
**Quitter**	0.914 (0.789-1.059)	1.228 (1.046-1.441)
**Current smoker**	1.102 (0.997-1.218)	1.750 (1.551-1.973)
**All-cause death**	P=<0.001	P=<0.001
**Never smoker**	1 (reference)	1 (reference)
**Ex-smoker**	1.298 (1.215-1.387)	1.138 (1.049-1.236)
**Quitter**	1.473 (1.330-1.632)	1.831 (1.637-2.048)
**Current smoker**	1.226 (1.310-1.331)	1.895 (1.723-2.084)

Model 1: age and sex-adjusted

Abbreviation: CI, confidence interval; HR, hazard ratio.

Online Table S3. The proportion of patients treated with antiarrhythmic drugs and undergoing AF ablation

	Total (n=97,637)	Never smokers (n=50,036)	Ex-smokers (n=26,621)	Quitters (n=6768)	Current smokers (n=14,212)	p-value
**AAD use**	23,409 (24.0)	11,273 (22.5)	7328 (27.5)	1760 (26.0)	3048 (21.4)	<0.001
**Class Ic**	15,587 (16.0)	7613 (15.2)	4861 (18.3)	1033 (15.3)	2080 (14.6)	<0.001
**Class III**	8045 (8.2)	3763 (7.5)	2533 (9.5)	754 (11.1)	995 (7.0)	<0.001
**AF ablation**	1145 (1.2)	477 (1.0)	450 (1.7)	110 (1.6)	108 (0.8)	<0.001

AAD was defined as class Ic and III drugs.

Abbreviation: AAD, antiarrhythmic drugs; AF, atrial fibrillation.

Online Table S4. Hazard ratios of primary and secondary outcomes after additionally adjusting whether performing early rhythm control by antiarrhythmic drugs or AF catheter ablation

	HR (95% CI)	p-value	HR* (95% CI)	p-value
**Ischemic stroke**				
Never smoker	1 (reference)	<0.001	0.593 (0.525-0.671)	<0.001
Ex-smoker	1.022 (0.917-1.140)		0.607 (0.541-0.680)	
Quitter	1.191 (1.014-1.399)		0.707 (0.599-0.834)	
Current smoker	1.685 (1.491-1.904)		1 (reference)	
**All-cause death**				
Never smoker	1 (reference)	<0.001	0.585 (0.532-0.645)	<0.001
Ex-smoker	1.112 (1.024-1.206)		0.651 (0.595-0.711)	
Quitter	1.454 (1.299-1.628)		0.851 (0.756-0.958)	
Current smoker	1.708 (1.551-1.881)		1 (reference)	
**Fatal ischemic stroke**				
Never smoker	1 (reference)	<0.001	0.452 (0.335-0.612)	<0.001
Ex-smoker	1.063 (0.809-1.396)		0.481 (0.360-0.642)	
Quitter	1.014 (0.651-1.581)		0.459 (0.290-0.726)	
Current smoker	2.211 (1.635-2.989)		1 (reference)	
**Death from cerebrovascular events**				
Never smoker	1 (reference)	<0.001	0.521 (0.400-0.678)	<0.001
Ex-smoker	1.071 (0.850-1.349)		0.558 (0.434-0.718)	
Quitter	1.290 (0.919-1.809)		0.672 (0.470-0.960)	
Current smoker	1.919 (1.475-2.497)		1 (reference)	

*HRs are presented the reference group as the current smoker. Abbreviation: AF, atrial fibrillation.

Online Table S5. The proportion of patients with obstructive sleep apnea

	Total (n=97,637)	Never smokers (n=50,036)	Ex-smokers (n=26,621)	Quitters (n=6768)	Current smokers (n=14,212)	p-value
**OSA**	361 (0.4)	133 (0.3)	125 (0.5)	37 (0.5)	66 (0.5)	<0.001

Abbreviation: OSA, obstructive sleep apnea.

Online Table S6. Hazard ratios of primary and secondary outcomes after additionally adjusting obstructive sleep apnea

	HR (95% CI)	p-value	HR* (95% CI)	p-value
**Ischemic stroke**				
Never smoker	1 (reference)	<0.001	0.591 (0.523-0.668)	<0.001
Ex-smoker	1.022 (0.916-1.139)		0.604 (0.539-0.677)	
Quitter	1.194 (1.017-1.403)		0.706 (0.599-0.832)	
Current smoker	1.692 (1.497-1.912)		1 (reference)	
**All-cause death**				
Never smoker	1 (reference)	<0.001	0.581 (0.528-0.640)	<0.001
Ex-smoker	1.110 (1.023-1.205)		0.645 (0.590-0.705)	
Quitter	1.455 (1.300-1.629)		0.846 (0.752-0.952)	
Current smoker	1.720 (1.562-1.894)		1 (reference)	
**Fatal ischemic stroke**				
Never smoker	1 (reference)	<0.001	0.449 (0.332-0.606)	<0.001
Ex-smoker	1.061 (0.808-1.394)		0.476 (0.357-0.635)	
Quitter	1.009 (0.647-1.573)		0.453 (0.286-0.717)	
Current smoker	2.229 (1.649-3.013)		1 (reference)	
**Death from cerebrovascular events**				
Never smoker	1 (reference)	<0.001	0.516 (0.396-0.671)	<0.001
Ex-smoker	1.068 (0.848-1.346)		0.551 (0.428-0.708)	
Quitter	1.285 (0.916-1.804)		0.663 (0.464-0.947)	
Current smoker	1.940 (1.491-2.523)		1 (reference)	

*HRs are presented the reference group as the current smoker

Online Figures

Online Figure S1. Hazard ratios for the risk of ischemic stroke and all-cause death in sequentially adjusted multivariable models

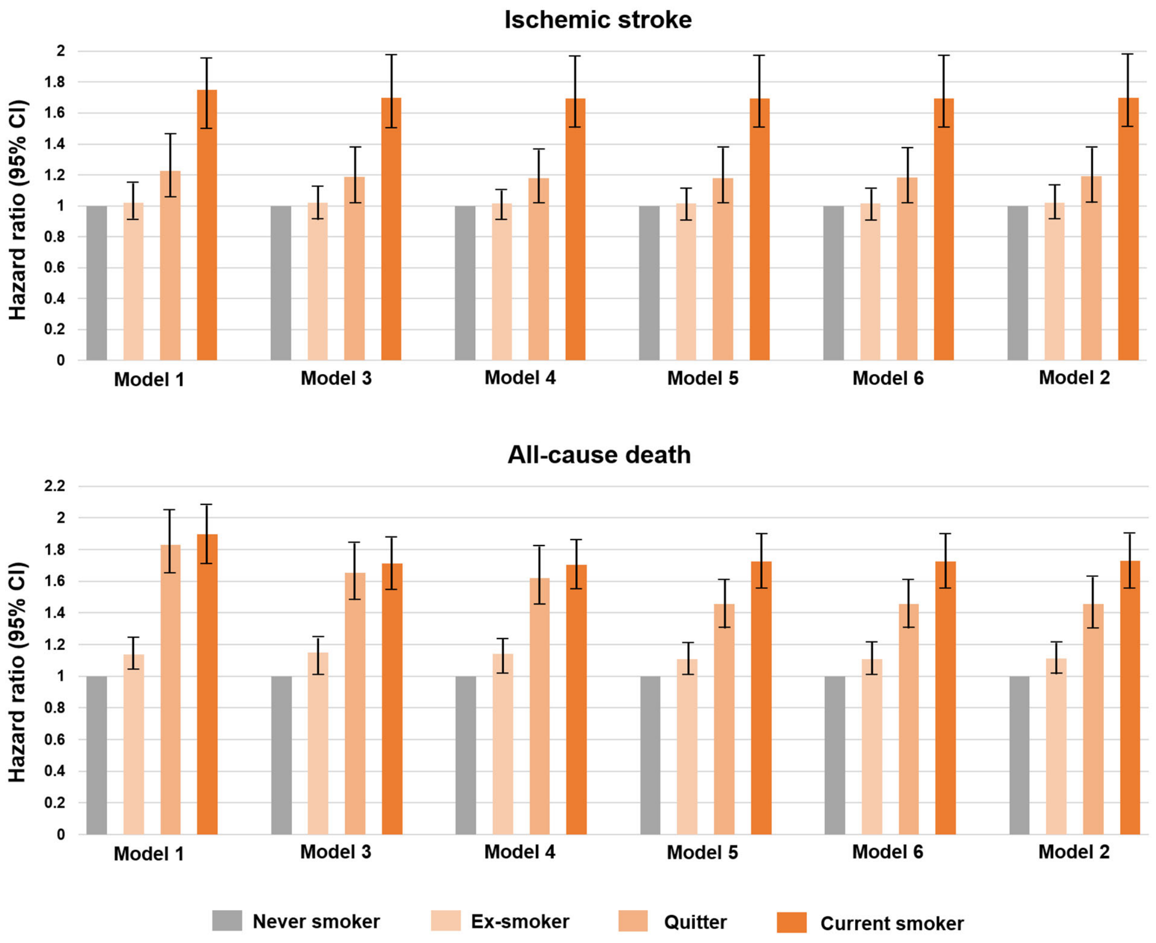

Abbreviation: CI, confidence interval.

Model 1: age and sex

Model 3: age, sex, baseline body mass index, heavy drinker, regular exercise, low income, and CHA_2DS_2-VASc score

Model 4: age, sex, baseline body mass index, heavy drinker, regular exercise, low income, comorbidities including hypertension, diabetes mellitus, dyslipidemia,

heart failure, prior myocardial infarction, and peripheral artery disease.

Model 5: model 4 + comorbidities including chronic kidney disease, chronic obstructive pulmonary disease and cancer.

Model 6: model 5 + CHA_2DS_2-VASc score

Model 2: model 6 + baseline medications including OACs, aspirin, $P2Y_{12}$ inhibitor and statin

Online Figure S2. Hazard ratios of smoking status on the risk of ischemic stroke and all-cause death in men and women

(A) Ischemic stroke

Group	Number	Events	IR	Model 2 HR (95% CI)		Model 2* HR (95% CI)	
**Men**				P<0.001	Pint=0.102	P<0.00`	Pint=0.102
**Never smoker**	15109	486	10.1	1 (reference)		0.612 (0.536-0.698)	
**Ex-smoker**	25941	774	9.6	0.993 (0.886-1.113)		0.607 (0.540-0.684)	
**Quitter**	6337	176	8.6	1.106 (0.929-1.318)		0.677 (0.569-0.806)	
**Current smoker**	13452	479	11.0	1.634 (1.433-1.864)		1 (reference)	
**Women**				P=0.001		P=0.00`	
**Never smoker**	34927	1121	10.0	1 (reference)		0.614 (0.420-0.898)	
**Ex-smoker**	680	21	10.7	1.154 (0.749-1.779)		0.709 (0.402-1.250)	
**Quitter**	431	24	18.1	1.908 (1.271-2.862)		1.172 (0.677-2.028)	
**Current smoker**	760	28	12.5	1.628 (1.113-2.380)		1 (reference)	

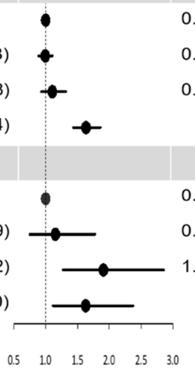

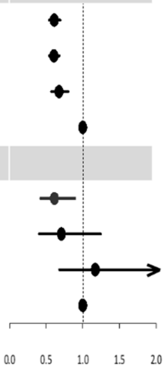

(B) All-cause death

Group	Number	Events	IR	Model 2 HR (95% CI)		Model 2* HR (95% CI)	
**Men**				P<0.001	Pint=0.016	P<0.001	Pint=0.016
**Never smoker**	15109	857	17.5	1 (reference)		0.630 (0.569-0.698)	
**Ex-smoker**	25941	1428	17.4	1.066 (0.980-1.161)		0.672 (0.613-0.737)	
**Quitter**	6337	409	19.7	1.378 (1.222-1.553)		0.869 (0.768-0.982)	
**Current smoker**	13452	729	16.3	1.586 (1.432-1.757)		1 (reference)	
**Women**				P<0.001		P<0.001	
**Never smoker**	34927	1344	11.7	1 (reference)		0.371 (0.278-0.495)	
**Ex-smoker**	680	35	17.4	1.477 (1.054-2.069)		0.548 (0.354-0.847)	
**Quitter**	431	31	22.5	1.508 (1.054-2.159)		0.559 (0.355-0.881)	
**Current smoker**	760	49	21.3	2.696 (2.022-3.596)		1 (reference)	

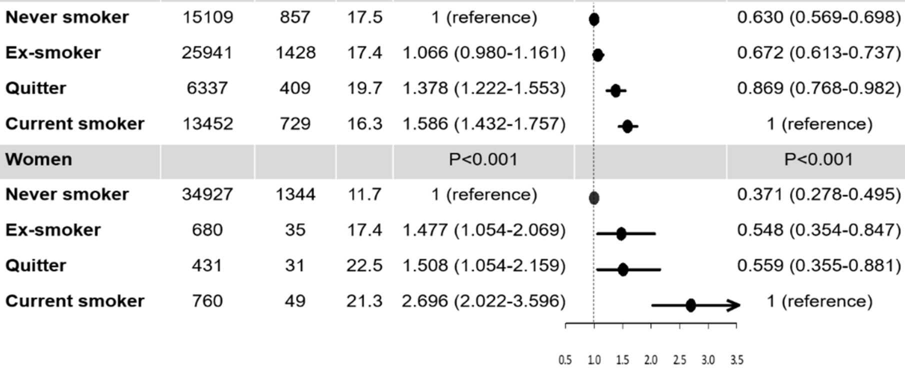

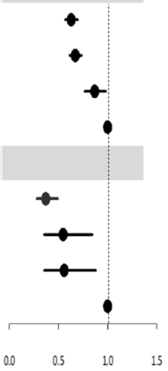

* With current smokers as the reference group

IR, per 1000 person-years

Abbreviation: CI, confidence interval; HR, hazard ratio; IR, incidence rate; Pint, p for interaction.

Online Figure S3. Subgroup analyses by age (<65 years, 65 to 74 years, and ≥75 years) and CHA_2DS_2-VASc score (<3 and ≥3)

(A) Age

Subgroup		Number	Events	IR	Model 2 HR (95% CI)		Model 2* HR (95% CI)	
**Ischemic stroke**						P_{int}<0.001		P_{int}<0.001
**<65 years**	**Never smoker**	24667	346	4.2	1 (reference)		0.509 (0.413-0.627)	
	**Ex-smoker**	14538	277	5.9	1.245 (1.013-1.530)		0.634 (0.533-0.753)	
	**Quitter**	4622	102	6.7	1.614 (1.249-2.086)		0.822 (0.652-1.035)	
	**Current smoker**	10908	262	7.3	1.964 (1.595-2.419)		1 (reference)	
**65-74 years**	**Never smoker**	16792	720	13.4	1 (reference)		0.577 (0.476-0.699)	
	**Ex-smoker**	8472	317	12.4	0.937 (0.791-1.110)		0.540 (0.450-0.649)	
	**Quitter**	1627	65	12.8	0.947 (0.721-1.245)		0.546 (0.411-0.725)	
	**Current smoker**	2567	187	23.4	1.734 (1.431-2.101)		1 (reference)	
**≥75 years**	**Never smoker**	8577	541	23.2	1 (reference)		0.771 (0.574-1.034)	
	**Ex-smoker**	3611	201	21.2	0.950 (0.777-1.163)		0.732 (0.545-0.983)	
	**Quitter**	519	33	23.2	1.027 (0.711-1.483)		0.791 (0.515-1.215)	
	**Current smoker**	737	58	28.8	1.298 (0.967-1.742)		1 (reference)	
**All-cause death**						P_{int}<0.001		P_{int}<0.001
**<65 years**	**Never smoker**	24667	317	3.8	1 (reference)		0.468 (0.382-0.573)	
	**Ex-smoker**	14538	286	6.0	1.254 (1.022-1.538)		0.586 (0.497-0.692)	
	**Quitter**	4622	149	9.6	1.759 (1.394-2.219)		0.823 (0.673-1.005)	
	**Current smoker**	10908	307	8.4	2.138 (1.746-2.617)		1 (reference)	
**65-74 years**	**Never smoker**	16792	813	14.6	1 (reference)		0.565 (0.483-0.659)	
	**Ex-smoker**	8472	598	22.8	1.181 (1.035-1.348)		0.667 (0.579-0.768)	
	**Quitter**	1627	188	36.0	1.598 (1.338-1.907)		0.902 (0.750-1.084)	
	**Current smoker**	2567	300	35.6	1.771 (1.517-2.068)		1 (reference)	
**≥75 years**	**Never smoker**	8577	1071	44.0	1 (reference)		0.700 (0.587-0.834)	
	**Ex-smoker**	3611	579	58.7	1.017 (0.899-1.149)		0.711 (0.599-0.845)	
	**Quitter**	519	103	69.3	1.080 (0.874-1.335)		0.756 (0.591-0.967)	
	**Current smoker**	737	171	80.4	1.429 (1.199-1.703)		1 (reference)	

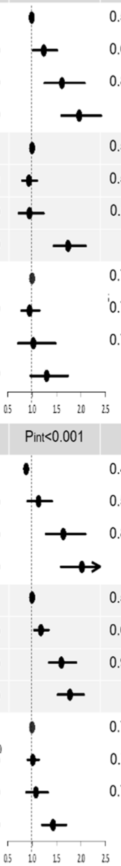
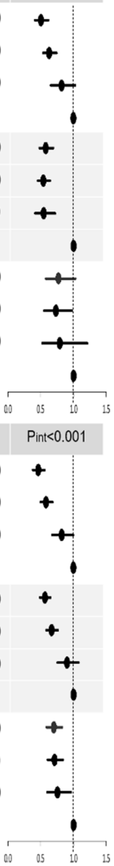

(B) CHA_2DS_2-VASc score

Subgroup		Number	Events	IR	Model 2 HR (95% CI)		Model 2* HR (95% CI)	
**Ischemic stroke**						P_{int}=0.027		P_{int}=0.027
**<3**	**Never smoker**	23842	403	5.0	1 (reference)		0.570 (0.480-0.677)	
	**Ex-smoker**	17770	410	7.1	1.052 (0.896-1.235)		0.599 (0.515-0.697)	
	**Quitter**	4631	96	6.2	1.139 (0.897-1.445)		0.649 (0.515-0.816)	
	**Current smoker**	11067	319	8.6	1.755 (1.478-2.085)		1 (reference)	
**≥3**	**Never smoker**	26194	1204	15.2	1 (reference)		0.626 (0.523-0.749)	
	**Ex-smoker**	8851	385	15.5	0.986 (0.849-1.144)		0.617 (0.517-0.736)	
	**Quitter**	2137	104	16.5	1.229 (0.987-1.530)		0.769 (0.605-0.979)	
	**Current smoker**	3145	188	21.0	1.598 (1.336-1.911)		1 (reference)	
**All-cause death**						P_{int}<0.001		P_{int}<0.001
**<3**	**Never smoker**	23842	430	5.3	1 (reference)		0.505 (0.433-0.589)	
	**Ex-smoker**	17770	559	9.6	1.198 (1.039-1.383)		0.605 (0.530-0.690)	
	**Quitter**	4631	208	13.3	1.774 (1.480-2.126)		0.895 (0.755-1.061)	
	**Current smoker**	11067	413	10.9	1.981 (1.698-2.312)		1 (reference)	
**≥3**	**Never smoker**	26194	1771	21.7	1 (reference)		0.638 (0.561-0.725)	
	**Ex-smoker**	8851	904	35.2	1.080 (0.977-1.195)		0.689 (0.609-0.780)	
	**Quitter**	2137	232	35.5	1.256 (1.082-1.457)		0.801 (0.679-0.945)	
	**Current smoker**	3145	365	39.2	1.568 (1.379-1.782)		1 (reference)	

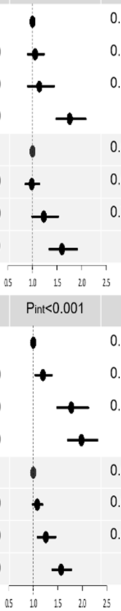
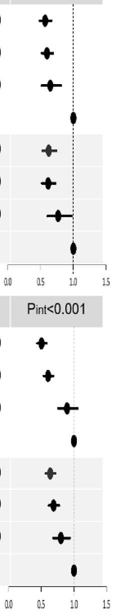

* With current smokers as the reference group

IR, per 1000 person-years

Abbreviation: CI, confidence interval; HR, hazard ratio; IR, incidence rate; P_{int}, p for interaction.
